# Malnutrition and Inadequate Eating Behaviour during Hospital Stay in Geriatrics—An Explorative Analyses of NutritionDay Data in Two Hospitals

**DOI:** 10.3390/nursrep11040085

**Published:** 2021-11-16

**Authors:** Fabian Graeb, Reinhold Wolke

**Affiliations:** Institute for Health Care and Nursing Sciences, Faculty of Social Work, Education and Nursing Sciences, Esslingen University, 73732 Esslingen, Germany; reinhold.wolke@hs-esslingen.de

**Keywords:** food intake, nutritional intervention, malnutrition screening, nutritionDay

## Abstract

(1) Background: Malnutrition in hospitalized patients is prevalent worldwide, but the severity of the issue is often underestimated by practitioners. The purpose of this study is to investigate the prevalence of malnutrition and inadequate eating behaviour in a geriatric sample. (2) Methods: Two hospitals participated with six wards on nutritionDay in 2017, 2018 and 2019. Nutritional status, food intake, and nutritional interventions were analyzed for all patients ≥ 65 years (*n* = 156), using the official nutritionDay questionnaires. Malnutrition risk is identified by Malnutrition Universal Screening Tool (MUST), malnutrition by the ESPEN criteria (European Society of Clinical Nutrition and Metabolism). (3) Results: According to MUST (*n* = 136) 16.9% (*n* = 23) were at medium risk of malnutrition, 33.8% (*n* = 46) at high risk of malnutrition, 28.1% (*n* = 38) were malnourished. Overall, 62.8% (*n* = 98) showed an inadequate eating behaviour during hospital stay. Moreover, patients with inadequate nutrition had significantly worse self-reported health statuses (*p* = 0.001; r = −0.276), were less able to walk on nutritionDay (*p* = 0.002; r = −0.255), had eaten little in the week before admission to hospital (*p* < 0.001; r = −0.313), and had an increased length of stay (*p* = 0.036; r = −0.174). (4) Conclusion: To identify malnourished patients is a significant barrier for practitioners seeking to administer specific, tailored interventions. Malnutrition screening protocols must be improved, just as nutrition monitoring in general.

## 1. Introduction

Prevalence rates of malnutrition in hospitals are high, varying between 19–40% depending on different cohorts and methods [1,2,3]. Geriatric patients have an even greater risk of malnutrition, with rates up to 56.2% [4,5,6]. Long-term care facilities report significant declines in the nutritional status of their residents after a hospitalization. Moreover, several studies show an increased mortality risk related to symptoms of malnutrition like underweight and reduced food intake, especially in the elderly [7,8,9,10]. In Addition, recent Studies during the current COVID-19 pandemic shows high prevalence rates for unintended weight loss, malnutrition risk and malnutrition in hospitalised patient [11] and significant associations of worsen nutritional state and mortality risk [12]. These factors are also associated with longer length of stay, and consequently, higher costs to the hospital and patient [1,6,13].

These studies describe an ongoing and highly underestimated problem. Due to the lack of adequate risk screenings, malnutrition often stays unrecognized [14]; Consequently, continuous and targeted nutrition management is difficult to achieve [15,16,17]. These realities in combination with the persistent deficits in clinical practice [1,18] prompted the development of a research project focused on the prevention and treatment of malnutrition in hospitalized geriatric patients. The overall goal of the research project, “Development, implementation and evaluation of a protocol for prevention and treatment of malnutrition in hospitalized geriatric patients”, was the development of an interdisciplinary nutrition protocol in two clinics in Stuttgart, Germany. With the help of a research-based practical protocol, malnutrition could be prevented or treated in a targeted manner, thereby avoiding serious health consequences for these patients [19,20].

The project started with a survey on nutritionDay 2017 and the following two years. In this worldwide annual survey, data about nutritional status, treatment, and nutrition management are collected through standardised questionnaires [21]. With the help of this data, many hypotheses on nutritional behaviour and causes of malnutrition have been explored by numerous researchers around the world. For example, one study showed that reduced meal intake during the week before hospitalization, confinement to bed, female sex, low body mass index, younger and older ages are strong, significant predictors of reduced food intake on nutritionDay [22]. As far as we know, a comparable evaluation with a focus on older patients has not been carried out yet.

The aim of this study is to get a closer look at malnutrition in patients 65 years or older who were being treated in the participating wards of the two project hospitals:

- Nutritional status of the included patients, prevalence of malnutrition risk, and malnutrition,
- indicators of inadequate eating behaviour during hospital stay, prevalence and associated factors of inadequate eating behaviour,
- nutritional support offered to the patients by staff and the question of who gets this support will be the focus in this explorative analysis.

## 2. Materials and Methods

NutritionDay takes place on first Thursday of November, every year. Hospitals and nursing homes worldwide are invited to participate on nutritionDay. This analysis contains data from six hospital wards, participating on nutritionDay in the years 2017, 2018 and 2019. The data were collected via questionnaire from 205 patients, which corresponds to approximately one third of all the patients in those wards on the nutritionDays. Of these 205 patients, 156 were 65 years or older. The following analyses utilized data from these 156 older patients. 

In all three years the official questionnaires of the nutrionDay were used. These questionnaires can be downloaded for free in multiple languages from the official nutritionDay website (www.nutritionday.org, accessed on 20 September 2021). For the following secondary analysis the questionnaires “2 a/b About your patient”, “3 a/b Patient questionnaire” and “Outcome Evaluation” were taken. “Questionnaire 2a/b (About your patient)” were answered by taking the information from the patient records (e.g., age, diagnosis at admission, comorbidities, surgery, nutrition state assessed by the staff, nutritional interventions etc.). The patients were interviewed by using the “3 a/b Patient questionnaire”. The Questions were about the overall health status (e.g., walking ability, medication prior to hospitalisation, seen a doctor/been admitted to hospital/night spent in hospital over the last 12 months), nutritional behaviour in general (dietary habits), nutritional intake in the week before admission (How well have you eaten—from more than normal to about a quarter to nearly nothing), eating on nutritionDay (e.g., need for help, portion size, interruption, how much eaten to lunch), and reasons for not eating all of the meal (e.g., did not like the smell/taste, chewing problems, normally eat less, was not allowed to, had an exam, surgery or test etc.). The Outcome Evaluation has been performed 30 days after nutritionDay and contains the discharge date (to calculate length of stay), outcome (e.g., still in the hospital, transferred to another hospital, death) and readmission since nutritionDay (no, yes, unknown). Further information’s are described in Table 1).

### 2.1. Nutritional Status

**Figure 1:**
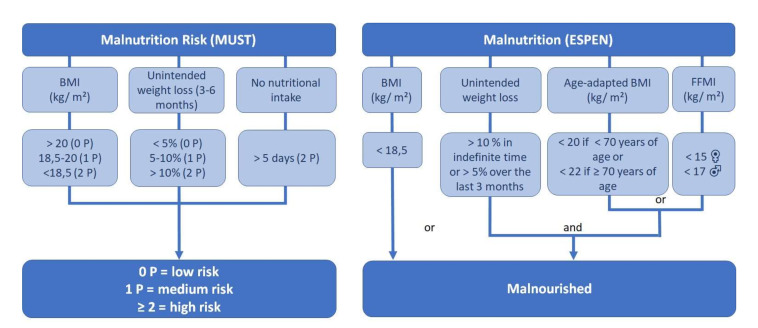
Malnutrition risk and Malnutrition [23,25].

Neither of the hospitals used a malnutrition screening protocol in their daily routine. So, patients at malnutrition risk had to been identified retrospectively. For this purpose, the nutritionDay data were analysed using the Malnutrition Universal Screening Tool (MUST). This screening tool contains the risk factors low BMI, unintended weight loss in 3–6 months and no nutritional intake for over 5 days [23]. So, the tool fits perfect to the existing nutritionDay data. Furthermore, Poulia et al. found a very strong correlation between MUST and ESPEN criteria for the definition of malnutrition [24], which is also used in this paper. The screening groups patients into three categories (Figure 1), based on the calculated score: Low (0 P), medium (1 P), and high (≥2 P) malnutrition risk. After identifying patients on medium or high malnutrition risk, according to ESPEN (European Society of Clinical Nutrition and Metabolism) guidelines, a second assessment has to be performed to identify already malnourished patients. For this purpose, the consensus-based ESPEN diagnostic criteria were used. It contains a low BMI (<18.5 kg/m^2^) or an unintended weight loss combined (>5%/3 months or <10%/indefinite time) with either an age adapted low BMI (<20 kg/m^2^ if <70 years; <22 kg/m^2^ if ≤70 years) or low muscle mass, defined as low fat free mass index (FFMI < 15 kg/m^2^ women, <17 kg/^2^ men) measured by bioelectrical bio-impedance analyses (BIA) [25].

### 2.2. Inadequate Eating Behaviour

Beside a worsened nutritional status at admission, monitoring the nutrition intake is one of the major methods to detect a malnutrition risk during hospital stay. It is also necessary to evaluate nutritional interventions. With the help of the answered questionnaires, it is possible to describe the nutritional status at admission, eating behaviour in the week prior to admission, and eating behaviour on nutritionDay. Therefore, changes and abnormalities in eating behaviour can be described for the sample. Depending on the specified questions of the nutritionDay instruments, the indicators of inadequate eating behaviour during the hospital stay were defined as:

- Eating less than before admission (the week before hospitalization)
- Eating the same as before admission if the intake had already been reduced
- Not eating the whole meal on nutritionDay and the reason is not “I normally eat less than served”
- Not eating on nutritionDay and the reason is not “I had an exam, surgery or test or I was not allowed to eat”

### 2.3. Data Analysis and Informed Consent

Statistical analyses were performed using SPSS 24^®^. Sample characteristics are described by percentage and total numbers, Median (Mdn) and Interquartil range (IQR). Mann–Whitney U Test and chi^2^ tests were performed to compare the groups adequate and inadequate eating behaviour. A *p* score of <0.05 is interpreted as a significant result. The effect size (r) is also specified: r ≥ 0.1 weak effect, r ≥ 0.3 fair effect and r ≥ 0.5 strong effect.

The study has been approved by the ethics committee of the German Society of Nursing Sciences in Witten (DGP, no. 17-005). Informed consent was obtained from all participants or their curator.

## 3. Results

Table 2 shows key characteristics of the sample such as sex, age, length of stay, admission diagnosis, comorbidities, unit specialty, and outcome after four weeks of hospitalization. The median age was 79.0 years (IQR 13.0), and there were more women (55.8%; *n* = 87). Chronic diseases were common among participants. Only 15.4% (*n* = 24) didn’t have any, while 35.3% (*n* = 55) had three or more, with cardiac insufficiency being the most frequent (24.4%; *n* = 38).

### 3.1. Health and Nutrition Status

The amount of prescribed medication can be read as a sign of high morbidity in the cohort. On nutritionDay, actually 69.9% (*n* = 109) were prescribed more than five different medications. The median number of prescribed drugs was 7.9 (±3.7). In the questionnaire, patients were asked how many times they had seen a doctor or been admitted to a hospital during the 12 months prior to their current hospital stay. Table 3 shows the answers and describes a cohort that had to use healthcare frequently. Overall, the patients had seen a doctor 4 times (IQR 4.0) in the last 12 months. On one side 9.6% (*n* = 15) did not have seen a doctor in this period, but on the other side 14.7% (*n* = 23) visited a doctor 12 times or even more often. One third had not been admitted to a hospital within the last year (33.3%, *n* = 52), but the majority had one or more hospital stays, concentrated around 1–3 nights in the last year (51.3%, *n* = 80). There were 23 patients (14.7%) that had spent ≥ 30 nights in hospital in the previous year. The overall median was 7.0 nights spent in hospital in the last year (IQR 20.0).

The patients were also asked to provide information on their self-reported health status (Table 3). In addition a minority of patients described their health as good or very good (24.4%; *n* = 38), while many more rated their health as poor or even very poor (37.2%, *n* = 58). The largest response category was patients reporting a fair health status (37.8%, *n* = 59).

Most of the patients lived at home before admission (80.1%; *n* = 125), suggesting relative independence. However, only 43.6% (*n* = 68) were able to walk without assistance from a person, walker, cane, or crutches. Table 3 shows the levels of walking abilities in general. Only a minority was not able to walk, with 9% (*n* = 14) in a wheelchair and 5.1% (*n* = 8) bedridden. On nutritionDay, 34.6% (*n* = 54) needed help by a person or walker, 11.5% (*n* = 18) were staying in bed. The walking abilities of the participants in hospital did not differ substantially from their general walking ability.

### 3.2. Nutritional Status

The median weight was 69.1 kg (IQR 22.2) and the median BMI 24.2 kg/m^2^ (IQR 6.7). Data in weight was only recorded in 85.9% (*n* = 134) and BMI in 80.1% (*n* = 125) of the patient on nutritionDay.

The majority of patients had no special dietary habits (80.8%, *n* = 126). Only five participants (3.2%) identified themselves as vegetarian and one (0.6%) reported a vegan diet. Intolerances or allergies were rare: only two patients (1.3%) reported lactose intolerance, and no other intolerances or allergies were mentioned.

A high proportion of the participants reported a significant reduction in food intake during the week prior to admission (Table 3). One quarter ate between nothing and about half of their normal volume (25%, *n* = 39). In the previous 3 months, 47.4% (*n* = 74) suffered from unintended weight loss, on average 6.0 kg (IQR 7.0; *n* = 61) or 10.3% (IQR 8.8; *n* = 54) of their body weight. According to MUST, 16.9% (*n* = 23) were at medium risk of malnutrition, and 33.8% (*n* = 46) were at high risk of malnutrition (*n* = 136). According to ESPEN guidelines, 28.1% (*n* = 38) were malnourished, whereas the patient records only identified 8.3% (*n* = 13) to be at risk of malnutrition and another 8.3% (*n* = 13) as malnourished (*n* = 135).

Nearly half of the patients ate only one half or less at lunch on nutritionDay (49.3%, *n* = 77). Out of these patients, 23.7% (*n* = 37) cited they “did not have their usual appetite” as a reason for not eating the whole meal, the most common response. There were 29 patients (18.6%) that stated that they “normally eat less than what was served”, and 22 (14.1%) claimed they were “not hungry at that time”. Only 1.3% (*n* = 2) of patients were “not allowed to eat”, and 3.2% (*n* = 5) missed the meal because of “an examination, surgery or test”. Overall, 62.8% (*n* = 98) demonstrated inadequate eating behaviour. Only 9.0% (*n* = 14) were dissatisfied or even very dissatisfied with the hospital food, which means that the majority (66.0%, *n* = 103) were partly satisfied or even very satisfied.

Out of all participating patients, 19.2% (*n* = 30) received at least one nutritional intervention, which included fortified/enriched hospital food, protein/energy supplement, a special diet, a record of food/nutrition intake, a nutrition treatment plan, and/or a nutrition expert was consulted. Table 4 shows a significant association between interventions and nutritional status. Being at high risk of malnutrition (MUST) or having a BMI < 18.5 kg/m^2^ made patients more likely to receive an intervention. On the other hand, 71.7% (*n* = 33) of the high-risk patients, 68.4% (*n* = 13) of those with a BMI < 18.5 kg/m^2^, and 76.3% (*n* = 29) of the malnourished patients did not receive any intervention. Of those that were identified as malnourished by the staff, more than two thirds received a nutritional intervention (69.2%; *n* = 9; *p* < 0.001; r = 0.453).

### 3.3. Associations with Inadequate Eating Behaviour

Comparing the groups with inadequate eating behaviour vs. adequate eating behaviour, Chi^2^-test showed no significant differences in sex, admission diagnosis, most comorbidities, surgery, ICU treatment, terminal illness, malnutrition risk, or malnutrition. However, in patients with Diabetes (77.1% vs. 58.7%; *p* = 0.047; r = 0.159), Infection (78.8% vs. 58.5%; *p* = 0.033; r = 0.171) and Other chronic diseases (79.6% vs. 55.1%; *p* = 0.003; r = 0.235), the ratio of patients with inadequate eating behaviour is significantly higher.

The Mann–Whitney U test (Table 5) showed very few significant associations. However, patients with inadequate eating behaviour reported worse health statuses (*p* = 0.001; r = −0.276), difficulty walking on nutritionDay (*p* = 0.002; r = −0.255), reduced food intake in the week before admission (*p* < 0.001; r = −0.313) and had an increased hospital stay (*p* = 0.036; r = −0.174). Though not statistically significant, there was a trend that suggested that patients with inadequate eating behaviour also tended to be older, have lower weight and BMI, and had lost weight in the previous three months, suggesting they were already in a state of reduced nutritional status.

## 4. Discussion

Prevalence of medium or high malnutrition risk (50.7%; *n* = 69) and malnutrition (28.1%; *n* = 38) were high in this sample. We also found several records with missing data on weight (14.1%; *n* = 22) and BMI (19.9%; *n* = 31). The problem of missing anthropometric data has been shown before in other nutritionDay publications. For example, Henriksen et al. reported BMI for only 66% of their sample [1]. In response to this problem, we offered the staff alternatives to measure body height and BMI by taking the knee height [26] or the circumference of the middle upper arm [27]. By this way, missing BMI data should be reduced in future nutritionDay surveys.

Furthermore, the ESPEN recommends a second assessment after the initial malnutrition screening to differentiate malnutrition risk from malnutrition. This contains the criteria low BMI (<18.5 kg/ m^2^) or age adapted BMI (<22 kg/m^2^ for people ≥70), in combination with either extending unintended weight loss or low fat free mass index (FFMI) respectively [25]. An ongoing problem with these criteria is that measuring the FFMI is not a common procedure in hospitals aside from scientific surveys. Although many hospitals have the facilities to measure FFMI through bioelectrical impedance analyses (BIA). It stays unclear why these resources are not used to measure body composition in patients who are at risk of malnutrition. This fact is another example for the big gap between scientific knowledge and daily clinical practice, related to the management of malnutrition.

### 4.1. The Undetected Malnutrition and Inadequate Feeding Behaviour

The low proportion of identified malnourished or malnutrition-risk patients emphasizes our thesis of the unappreciated role of malnutrition in clinical practice. However, to identify patients on malnutrition risk is the basis for a further assessment like the ESPEN criteria and nutritional interventions. The analysis of the nutritionDay data of German hospitals shows these problems as a widespread issue. Volkert et al. (2019) found that only 54.2% of the patients are weighted after admission and 9% do not have a BMI in their patient records. According to this analysis, 15% are moderately and 20% severely malnourished. However, the staff only identified 11.5% of patients to be at risk for malnutrition and another 11.6% as malnourished [28]. So, actual prevalence of malnutrition and the proportion of already identified patients by staff are comparable in this study. The wards of both participating hospitals implemented a malnutrition-risk screening after nutritionDay 2018. One hospital decided to integrate the screening tool into the nursing records, and in the other hospital physicians were made responsible for malnutrition screening. The nutritionDay data of 2019 shows ongoing difficulties by using the screening tool. Reasons are unknown and would be just speculative graded. A survey by Eglseer at al. for example showed improved screening rates (83.5%) for the first month after implementation, but three months later the staff only completed a nutritional screening for 4.6% of the newly admitted patients. Major barriers to the use of the screening tool were missing weight and weight loss data, deficient support by the management, and specific problems with some parts of the screening tool [29]. So, evidently, it is very difficult to integrate these screenings into the staff’s everyday routine and must be attended over a longer time.

In general malnutrition is rarely diagnosed. This has been proved in a former study by analysing the discharge letters of nursing home residents after a hospital stay of at least three days. Only 1.5% were marked as malnourished. However, retrospective data analyses showed that 38% were at malnutrition risk and 19.8% were already malnourished at admission. Upon discharge, these rates climbed up to 48.6% and 24.6% respectively [30]. The underestimation of malnutrition is also seen in clinical routine data. For example, the Quality in Medicine Initiative (IQM = Initiative Qualitätsmedizin) reported an average diagnosis of sever malnutrition in only 1.1% of the patients aged 65 years and older [31]. Apart from the issue with inadequate screening, it has to be mentioned that the ICD-10 definition of malnutrition does not have anything in common with scientific based diagnostic criteria [32].

Beyond identification, previously diagnosed inadequate eating behaviour was not getting enough attention from the nurses and physicians in the two participating hospitals. Only two thirds of the patients identified as malnourished received nutritional therapy or an intervention. However, it is not known how often patients declined an intervention, because this is probably not noted in the patient records. The main reason for the mostly unrecognized inadequate eating behaviour is probably the little attention on nutrition care in general. A lack of proper education in healthcare professionals, no standardised nutrition care process, and the short hospital length of stay has been identified as barriers for a good nutritional care in former studies [14]. In context of the educational problem, it has to be considered that geriatrics with obesity could also suffer from malnutrition risk [33], while being overweight and obesity doesn’t modify the negative effect of weight loss on in-hospital mortality [34]. In fact, there are several pathological pathways in obese persons that lead to low muscle mass. In addition, these pathways like inflammation or insulin resistance are “typical” on obesity [35]. Unintended weight loss in geriatrics could mean muscle loss and is probably even more often unrecognized in overweighted or obese patients. Furthermore, geriatrics need an increased protein intake to compensate anabolic resistance, especially in obese geriatrics [36]. So, eating less automatically leads to deficient protein intake, which also leads to weight loss and loss of muscle mass. A low muscle mass is associated with negative outcome such as poor wound healing after surgery, increased risk of readmission, falls, longer hospital stay and mortality [37]. For these reasons, it’s necessary to evaluate nutritional state (especially weight loss) and eating behaviour during hospital stay in geriatrics, as well in patients with obesity.

The focused few on patients with inadequate eating behaviour during their hospital stay suggests an overall decline in health and nutritional status in this group. The apparent differences by higher age, more frequent doctor visits and/or being hospitalized for more than a day in the last 12 months, and more chronic diseases indicate a worsened health status. In addition, the trend in lower weight, BMI, extended weight loss in the previous three months, and a higher MUST Score—although not statistically significant—suggest an already declined nutritional state. This illustrates once again the need to conduct malnutrition screening at admission and to use a nutritional protocol for those who are malnourished or at risk of malnutrition, as required in the current ESPEN guidelines [38].

Interestingly, comorbidities didn’t show many significant differences. The sample had a wide range of admission diagnoses, and with only 156 participants, it could be too small to expect significant associations. Nevertheless, inadequate eating behaviour was significantly more frequent in patients with Diabetes, Infection and Other chronic disease. Other studies have shown an increased level of inflammation parameters like CRP in malnourished patients [39]. In addition, pronounced inflammation seems to decrease the success of nutritional interventions [40]. Fatigue for example, as a common side effect of infection and inflammation, leads to a reduced appetite. Unfortunately, serum marker for an ongoing or acute inflammation are not part of the nutritionDay instruments. But the association of Infection and inadequate eating behaviour in this study implies inflammation as a possible risk factor for reduced food intake.

Schindler et al. analysed nutritionDay Data of patients worldwide and of all ages. Their findings are partly comparable: reduced intake on nutritionDay is associated with reduced intake in the week before admission, female sex, younger and older age and low BMI [22]. However, logistic regression of patients who ate a full meal on nutritionDay did not include chronic diseases and infection as risk factors.

Overall, patients with inadequate eating behaviour had an increased length of stay. It is most likely that there is also a link to the individual’s health state. But it can’t be clarified if a reduced nutritional intake is causing a general reduced health status, or if acute diseases or increasing health problems are leading to a reduced intake.

### 4.2. Limitations

The major limitation of this research is the comparatively small sample. This makes it difficult to perform more robust statistical tests. For this reason, further research is needed. It would be reasonable to analyse the yearly growing nutritionDay database for subgroups, like geriatric patients. In this way, risk factors for reduced food intake could be identified, especially for geriatric patients. Further it would be interesting to compare eating behaviour in hospitals and risk factors for reduced intake in younger and older patients or between geriatrics living at home and in nursing-homes. The second significant limitation is the fact that the shown data are based on a secondary analysis. The patient identifiers were kept confidential before data analysis. This makes it impossible to add data from the patient records in retrospect like serum marker of inflammation. The latest international consensus for the diagnosis of malnutrition, the GLIM criteria, also includes the ESPEN criteria as Phenotypic criteria and, in addition, Reduced Food Intake or Assimilation and Inflammation as Etiologic Criteria [41]. This definition has been released in 2019, so markers of inflammation like serum C-reactive protein (CRP) or serum cytokine were not collected, and as a result, the GLIM criteria is not used in this analysis.

## 5. Conclusions

The study shows the supposed underestimated role of malnutrition in clinical practise. This points the need for an increased intention on the nutritional status of hospitalised patients. Moreover, nutritional interventions like the EFFORT study have shown strong effects on clinical outcome of malnourished patients, even in geriatrics [42,43]. So, it is necessary to detect malnutrition and feeding related difficulties much more reliably. For this purpose, malnutrition screening is already essential. Furthermore, nurses and auxiliary nurses are in a key position to detect daily problems like reduced food intake during hospital stay. This ongoing observation is also necessary for an individualised nutritional support. However, knowledge and sensitivity for nutrition-related problems in daily care must been educated. This study shows also participating on nutritionDay as an useful instrument for quality control in nutritional support.

## Figures and Tables

**Table 1:** Used nutritionDay Questionnaire.

Questionnaire	Asked Variables
2 a/b About your patient (taken from the patient files)	year of birth, sex
	weight/ height at admission
	diagnosis at admission (by ICD-10 maingroups), main reason for admission, comorbidities (e. a. cancer, dementia)
	surgery during hospital stay, admission to Intensive Care Unit (ICU), number of different medications, identified as malnourished or at risk of malnutrition
	nutrition intake/diet (e.g., regular food, special diets), nutritional interventions (e.g., recording nutrition intake, developing a nutrition treatment plan)
3 a/b Patient questionnaire (by interviewing the patients)	typical dietary habits (e.g., no special habits, vegetarian, gluten-free diet)
	living before hospital admission, general ability to walk, subjective health state in general
	contact with doctor and hospital stays in the last 12 months, numbers of daily different medications (prior to hospital stay)
	weight five years ago, weight lost within the last three months (if yes, amount of kg), get any nutritional information or intervention
	eating in the week before admission, satisfaction with the hospital food
	Today: getting help with eating, eating interruption, ordered portion size, how much ate for lunch, reasons for eating less/nothing
	changed food intake since admission, todays subjective condition, walking ability today
Outcome Evaluation (taken from the patient files)	30 days after nutritionDay: discharge date, discharge diagnosis, outcome (e.g., still in hospital, rehabilitation, death)

**Table 2:** Sample characteristics.

Variable	*n*		Mdn	(IQR)	%	(*n*)
Age	156		79.0	(13.0)		
Sex	156	male			41.7	(65)
		female			55.8	(87)
Length of stay (days)	156		12.0	(11)		
Admission diagnosis, ICD-10 groups	156	Respiratory system			18.6	(29)
		Musculoskeletal system and connective tissue			17.9	(28)
		Nervous system			15.4	(24)
		Circulatory system			10.3	(16)
Admission	156	Emergency			68.6	(107)
Comorbidities	156	Cardiac insufficiency			24.4	(38)
		Diabetes			22.4	(35)
		Cancer			22.4	(35)
		Chronic kidney disease			21.8	(34)
		Peripheral vascular disease			21.2	(33)
		Chronic lung disease			18.6	(29)
		Dementia			16.7	(26)
Unit Specialty	156	Internal Medicine/Geriatrics			35.9	(56)
		Internal Medicine/General			21.8	(34)
		Surgery/Orthopedic			16.0	(25)
		Neurology			20.5	(32)
		Interdisciplinary (Surgery/Internal)			5.8	(9)
ICU stay (≥1 day)	156				13.5	(21)
Surgery (any)	156				14.8	(23)
Outcome after 4 weeks	156	Still in the hospital/transferred to another hospital			10.3	(16)
		Transferred to long term care			20.5	(32)
		Rehabilitation			8.3	(23)
		Discharged home			59.6	(93)
		Death			1.3	(2)

ICD = International Classification of Diseases; ICU = Intensive Care Unit; IQR = Interquartile Range; Mdn = Median.

**Table 3:** Health and nutrition status.

Variable	*n*		Mdn	(IQR)	%	(*n*)
Living before admission	156	at home			80.1	(125)
		in nursing home or other live-in facilities			14.1	(22)
		Other/no answers			5.8	(9)
General ability to walk	156	no information			0.6	(1)
		No, I’m bedridden			5.1	(8)
		No, I have a wheelchair			9.0	(14)
		Yes, using a cane, walker or crutches			26.9	(42)
		Yes, with someone’s help			14.7	(23)
		Yes			43.6	(68)
Ability to walk today	156	no information			9.0	(14)
		No, I’m bedridden			11.5	(18)
		No, only with assistance			34.6	(54)
		Yes			44.9	(70)
Using health care over the last 12 months	144	Seen a doctor	4.0	(4.0)		
	150	been admitted to hospital	1.0	(2.0)		
	138	nights spent in hospital	7.0	(20.0)		
prescribed medication	156	on nutritionDay	8.0	(5.0)		
Self-reported health status	156	no information			0.6	(1)
		very poor			7.1	(11)
		poor			30.1	(47)
		fair			37.8	(59)
		good			22.4	(35)
		very good			1.9	(3)
Eating behaviour before admission	156	no information			3.2	(5)
		more than normal			1.9	(3)
		normal			59.6	(93)
		¾ of normal			10.3	(16)
		½ of normal			16.0	(25)
		¼ of normal to nothing			9.0	(14)
Eating behaviour on nutritionDay	156	no information			1.9	(3)
		all			48.1	(75)
		¾ portion			0.6	(1)
		½ portion			33.3	(52)
		¼ portion			10.3	(16)
		nothing			5.8	(9)
Satisfaction with offered hospital food	156	no information			7.1	(11)
		very/somewhat satisfied			66.0	(103)
		neutral			17.9	(28)
		dissatisfied/very dissatisfied			9.0	(14)
Weight in kg	134		69.1	(22.2)		
BMI in kg/m^2^	125		24.2	(6.7)		
Unintended weight lost	61	last 3 months, kg	6.0	(7.0)		
	54	last 3 months, % of body weight	10.3	(8.8)		
Malnutrition risk (MUST)	136	medium risk			16.9	(23)
		high risk			33.8	(46)
Malnourished	136	ESPEN guidelines			28.1	(38)

BMI = Body Mass Index; ESPEN = European Society of Clinical Nutrition and Metabolism; IQR = Interquartile Range; Mdn = Median; MUST = Malnutrition Universal Screening Tool.

**Table 4:** Nutritional status and nutritional intervention (Chi^2^-test).

	*n*		Get Any Nutritional Intervention		*p*	r
			%	(*n*)		
MUST	136	Low risk	10.4	(7)	0.023	0.235
		Medium risk	8.7	(2)		
		High risk	28.3	(13)		
Malnutrition ESPEN	135	Not malnourished	12.4	(12)	0.103	
		Malnourished	23.7	(9)		
BMI < 18.5 kg/m^2^	135	BMI ≥ 18.5 kg/m^2^	12.9	(15)	0.038	0.179
		BMI < 18.5 kg/m^2^	31.6	(6)		
Inadequate eating behaviour	156	No	12.1	(6)	0.081	
		Yes	23.5	(23)		
Malnourished, identified by staff	156	not malnourished	11.5	(15)	<0.001	0.453
		At risk	46.2	(6)		
		malnourished	69.2	(9)		

BMI = Body Mass Index; ESPEN = European Society of Clinical Nutrition and Metabolism; MUST = Malnutrition Universal Screening Tool; *p* = correlation; r = effect size.

**Table 5:** Group comparison inadequate food intake (Mann-Whitney-U test).

	*n*	Mdn (IQR)		*p*	r
		Inadequate	Not Inadequate		
Age	98/58	79.5 (12.3)	77.0 (15)	0.144	
Weight	83/51	67.8 (21.1)	71.0 (25.4)	0.608	
BMI	79/46	23.8 (6.5)	24.8 (8.2)	0.868	
Eating before admission	98/58	4.0 (2.0)	4.0 (0.0)	<0.001	−0.313
MUST Score	85/51	1.0 (2.0)	0.0 (2.0)	0.079	
Number of different medications (on nutritionDay)	98/58	8.0 (5.0)	8.0 (6.0)	0.775	
Self reported health	98/58	3.0 (1.0)	3.0 (2.0)	0.001	−0.276
Times seen a doctor	88/56	4.0 (3.7)	3.5 (7.5)	0.650	
Times been in hospital	95/55	1.0 (2.0)	1.0 (2.0)	0.555	
Number of nights in hospital	84/54	7.0 (21.0)	4.5 (17.8)	0.302	
Number of chronic diseases	98/58	2.0 (2.0)	2.0 (2.2)	0.121	
Unintended weight loss last 3 months (kg)	46/15	6.0 (7.0)	5.0 (3.0)	0.480	
Unintended weight loss last 3 months (%)	40/14	10.6 (6.8)	8.4 (7.3)	0.407	
Satisfaction with the offered meal	98/58	4.0 (2.0)	4.0 (1.0)	0.175	
Length of stay	92/53	14.0 (11.0)	10.0 (11.5)	0.036	−0.174
General walking ability	97/58	2.0 (2.0)	2.0 (2.0)	0.422	
Walking ability today	90/54	2.0 (1.0)	1.0 (1.0)	0.002	−0.255

BMI = Body Mass Index; IQR = Interquartile Range; Mdn = Median; MUST = Malnutrition Universal Screening Tool; *p* = correlation; r = effect size.
